# Synthesis, characterization, optical properties, biological activity and theoretical studies of a 4 nitrobenzylidene) amino) phenyl)imino)methyl)naphthalen-2-ol -based fluorescent Schiff base

**DOI:** 10.1016/j.heliyon.2024.e26349

**Published:** 2024-02-19

**Authors:** Sadeq M. AlHazmy, Mohamed Oussama Zouaghi, Ahmed N. Al-Hakimi, Thamer Alorini, Ibrahim A. Alhagri, Youssef Arfaoui, Rania Al-Ashwal, Lamjed Mansour, Naceur Hamdi

**Affiliations:** a Department of Chemistry, College of Science, Qassim University, Buraidah, 51452, Saudi Arabia; b Research Laboratory of Environmental Sciences and Technologies (LR16ES09), Higher Institute of Environmental Sciences and Technology, University of Carthage, Hammam-Lif, Tunisia; c School of Biomedical Engineering and Health Sciences, Faculty of Engineering, Universiti Teknologi Malaysia, Johor Bahru, 81310, Malaysia; d Advanced Diagnostic and Progressive Human Care Research Group, School of Biomedical Engineering and Health Science Teknologi Malaysia, Johor Bahru, 81310, Malaysia; e Zoology Department, College of Science, King Saud University, Saudi Arabia, P.O. Box 2455, Riyadh, 11451, Saudi Arabia; f Laboratory of Characterizations, Applications & Modeling of Materials (LR18ES08), Department of Chemistry, Faculty of Sciences, University of Tunis El Manar, 2092, Tunis, Tunisia

**Keywords:** Fluorescence, DFT calculations, Biological evaluation, Molecular docking, Quenching, Chemisensors

## Abstract

A new Schiff base, 1-(E)-(4-((E) 4nitrobenzylidene) amino) phenyl)imino) methyl)naphthalen-2-ol (4NMN), was prepared from the reaction of *p*-phenylenediamine with 2-hydroxy-1-naphthaldehyde and 4-nitrobenzaldehyde and characterized with spectroscopic analysis. UV-VIS and NMR. Frontier molecular orbitals, molecular electrostatic potential, and chemical reactivity descriptors of the synthesized compound were studied using molecular modeling methods. The antibacterial and antifungal activities of the Schiff base were studied for its minimum inhibitory concentration. The compound showed a higher effect on yeast than against bacteria. Density functional theory (DFT) calculations were performed to study the mechanism of reaction for the synthesis of 4NMN, and the results were consistent with the experimental findings. 4NMN exhibited moderate antibacterial and antifungal activities and demonstrated higher inhibition potential against different resistant strains compared to the reference drug gentamycin. The absorption and fluorescence spectra of 4NMN were measured in different solvents, and the effect of relative polarity and acidity on the medium was observed. An inner filter effect was observed at high concentrations, and the compound showed considerable fluorescence enhancement with increasing medium viscosity and fluorescence quenching by the addition of traces of Cr^1+^ and Cu^2+^. Additionally, molecular docking studies were conducted to investigate the efficiency of antibacterial and antifungal targets.

## 1 Introduction

Schiff bases and their metal complexes boast impressive biological activity against bacteria, fungi, and tumors [[1], [2], [3]]. They hold further promise as chemical nucleases for DNA binding, cleavage, and diagnostics [[4], [5], [6]]. Notably, o-hydroxy Schiff bases exhibit unique tautomerization due to phenol-imine and keto-amine hydrogen bonds [7,8], making them particularly interesting for future exploration. Heavy metals and other toxic cottings are serious healthy and environmental risks, making the development of trustworthy detection techniques necessary. In previous work, research on the methods and strategies of improving allergies and selective of fluorine chemical sensors with a Schiff base, which is specially manufactured to find heavy metals and toxic cottons [9]. This tautomerism induces changes in the electronic structure, making Schiff bases suitable for use as anion sensors. Their intramolecular hydrogen bond and proton transfer process enable this functionality [10]. Colorimetric sensors, leveraging hydrogen bonding, offer a simple and versatile approach to anion detection, requiring no fancy equipment [11,12]. Urea, porphyrins, and other hydrogen-bonding champs like amides, azophenols, oxadiazoles, benzoxazoles, and Schiff bases, have been explored as color-changing or light-emitting sensors for various anions. Numerous chemo-sensors have been investigated for their selectivity and sensitivity in recognizing and sensing anions [13]..

**Scheme 1:**
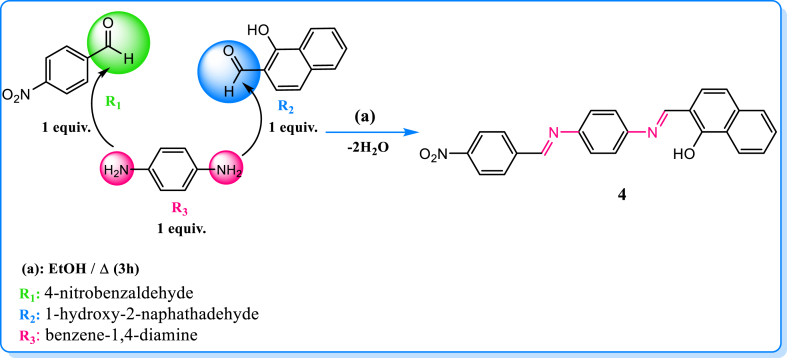
Synthesis of compound **4** via the reactants **R**_**1**_**, R**_**2**_and **R**_**3**_.

To this day, there have been no reported instances of the new Schiff base we synthesized, known as (E)-3,5-dimethoxy-2-((6-methoxybenzo[d]thiazol-2-ylimino)methyl)phenol, exhibiting remarkable sensitivity and selectivity (Scheme 1). The spectroscopic methods were employed to examine the structure of this Schiff base. Additionally, we utilized theoretical methods and DFT calculations at the B3LYP/6-31G+(d) level to investigate the possible formation mechanism of component 4 observed in experimental gas phase, implicit, and explicit models. Analysis of UV-vis and photoluminescence were performed on the aromatic salicylideneaniline some Schiff-base derivatives in a range of solvents. The effects of concentration on normal to tautomer ratios were observed, along with the influence of substituents and solvents on absorption and emission properties. There were notable variations in the extinction coefficient values in various solvents, highlighting the absorbance of the studied Sciff base earlier that is dependent on the solvent [14]. Furthermore, we measured the absorption and fluorescence spectra of 4NMN and calculated the quantum yields. The dye showed promising chemosensing efficiency, as the fluorescence efficiency decreased upon the addition of various low concentrations of ions. Previous studies have demonstrated that increasing the viscosity of the medium can enhance the fluorescence efficiency of certain flexible molecules. This observation led to the identification of a viscosity-dependent non-radiative mode [[15], [16], [17], [18], [19], [20]] as the internal conversion ($S_{1}^{*}$ →$S_{0}$) that decreases in viscous media, minimizing the stretching and twisting molecular motion in the excited state.

## 2 Experimental section

### 2.1 Instruments

#### 2.1.1 ^1^H and ^13^C NMR spectra

Proton-carbon NMR (^1^H and ^13^C NMR) spectroscopy measurements of the compound were performed by a Bruker spectrophotometer 850 MHz and 213 MHz, respectively, and using a CDCl_3_ solvent, TMS as a standard reference at a temperature of 25 °C, at Qassim University.

#### 2.1.2 UV-vis. and fluorescence spectra

Ultraviolet absorption measurement of the compound was carried out at a concentration 1 × 10^−4^ M using a Shimadzu spectrophotometer (UV-1650PC, Japan), in Dimethyl Sulfoxide (DMSO) as a solvent using a 1 cm quartz cell, wavelength range 200–800 nm. Steady-state measurements of the 4NMN dye and the different media were carried out using a JASCO FP-8200 spectrometer.

### 2.2 Synthesis of the component 4

The synthesis of the compound 1-(E)-(4-((E)-4-nitrobenzylidene)amino)phenyl)imino)methyl)naphthalen-2-ol, depicted in Scheme 1, was carried out in the following manner: A solution of 4-nitrobenzaldehyde (1.51 g, 0.01 mmol) in 20 mL ethanol and a solution of 1-hydroxy-2-naphathadehyde (1.72 g, 0.01 mmol) in 20 mL ethanol were combined with a solution of benzene-1,4-diamine (1.08 g, 0.01 mmol) in 20 mL ethanol. The resulting mixture was stirred using a magnetic stirrer at a temperature of 100 °C for a duration of 2 h. During this time, a dark red precipitate formed. The precipitate was subsequently filtered and subjected to TLC analysis, followed by multiple washes with hot ethanol. Colorsolid; Orang, yield = 82 %; mp 206 °C; ^1^H NMR *δ*_H_ (ppm): (d, *J* = 12 Hz, 6H, CH_3_), (m, 5H, H_arom_). ^13^C NMR *δ*_C_ (ppm): 163.22 (d, *J* = 227.18 Hz, C=O), 120.16–136.50(m, C_arom_), 54.87 (d, *J* = 6.80 Hz, MeO). IR (CHCl_3_, υ cm^−1^): (**C=N**) = 1615 (**C=C**) = 1590, .Anal. Calc. for C_24_H_17_N_3_O_3_ (395,42 g mol^−1^): C, 72.90; H, 4.33; N, 10.63; found C, 72.80, H, 4.25, N, 10.50%.

### 2.3 ^1^H NMR and ^13^C NMR spectra

The different spectrum values of the new compound showed agreement with the experimental results that were reached through the elemental analysis of the compound. On the other hand, the protons of the amine group disappeared from the new compound, indicating its association with the carbonyl group and the formation of a Schiff base. The spectrum showed one peak as a singlet at 12.30 ppm, which indicated a proton of the hydroxyl moiety, also the compound showed two bands at 10.80 and 10.00 ppm, which indicated the –N=CH– groups, because of the polarity of the hydroxyl group, which is nearer to the group whose proton occurs at 10.80 ppm, there are various shift between these protons. Within the spectrum of 6.65–8.10 ppm, a further signal that is attributed to aromatic protons occurred as a multiplet. On the other hand, the compound's ^13^C NMR revealed bands at 149.50, 148.40, and 147.77 ppm. The C–OH, –N=CH–, and –N=CH– groups, respectively, could be responsible for these bands. The limits for the aromatic carbons were placed in the 113.26–140.35 ppm range.

### 2.4 Photochemical stability

Using a modified A. J. Lees method that takes into consideration the decrease in absorbance at the excitation wavelength as photoirradiation time increases, photochemical quantum yields of 4NMN (ɸ_c_) were measured [21].

### 2.5 Fluorescence quantum yields

Depending on the emission wavelength range a dilute solution was used in the determination of the fluorescence efficiency, using either quinine sulfate or 9,10-diphenyle anthracene solutions. Ferrioxalate actinometry was used to determine the light intensity [22]. The fluorescence quantum yields were determined using Equation (1) below.

(1) $$\emptyset_{f}\left(s\right)=\emptyset_{f\;}\left(r\right)\times \frac{{\int I}_{s}}{{\int I}_{r}}\times \frac{A_{r}}{A_{s}}\times \frac{n_{s}^{2}}{n_{r}^{2}}$$

The integrals denote the corrected fluorescence peak areas, A and n denote the absorbance at the excitation wavelength, and the solvent's refractive index respectively. The subscripts s and r refer sample reference respectively.

### 2.6 Antimicrobial effect

The tests involving the antimicrobial activities of the compound were performed at the Yemen Standardization, using the popular standard method [[23], [24], [25], [26]].

### 2.7 Computational details

#### 2.7.1 DFT calculations

The Gaussian 16A suite of program [27] was utilized for all computations. Geometry optimizations were conducted without symmetry constraints using the B3LYP functional [28] and the 6-31G+(d) basis set [[29], [30], [31]]. To account for solvent effects, the implicit IEF-PCM solvation model [32] was employed. Frequency calculations at the same level of theory were performed to confirm the absence of imaginary frequencies, indicating local minima. Intrinsic reaction coordinate (IRC) calculations were employed to trace the reaction pathway and verify the energy profiles connecting each transition state (TS) to the associated reactant and product minima. For transition states, it was ensured that the structure possessed only one imaginary vibrational frequency [33].

The concept of molecular chemical reactivity is closely associated with theoretical chemistry, which is grounded in frontier molecular orbital theory. Density functional theory holds remarkable potential for obtaining the global reactivity descriptor, which provides insights into the overall behavior of molecules [34].

The electronic chemical potentials μ, chemical hardnesses η, and softnesses S of the investigated reactants were evaluated based on the one-electron energies of the frontier molecular orbitals, using the following equations [35].

(2) $$\mu =\left(E_{\text{HOMO}}+E_{\text{LUMO}}\right)/2$$

(3) $$\eta =E_{\text{LUMO}}-E_{\text{HOMO}}$$

(4) S=1/η

The values of μ and η were then used to calculate ω as follows:

(5) $$\omega =\mu^{2}/2\eta$$

Density Functional Theory (DFT) emerges as a powerful tool for examining the reactivity and selectivity of the investigated reaction [36], making it a suitable method for computing various chemical concepts such as electronegativity, electron affinity, ionization potential, and chemical potential. Moreover, it enables the determination of atomic descriptors used to assess the local reactive sites within a molecule [37]. Among these descriptors, the Fukui functions [38] are widely employed for understanding nucleophilic/electrophilic molecular reactivity. Yang and Mortier [39] proposed a procedure to compute the Fukui functions, utilizing the Mulliken population analysis and a finite-difference approximation to the *f*(r) function introduced by Parr and Yang [40]. This procedure can be expressed as:

(6) $$f_{k}^{+}=q_{k}\left(N+1\right)-\;q_{k}\left(N\right)$$

for nucleophilic attack

(7) $$f_{k}^{-}=q_{k}\left(N\right)-\;q_{k}\left(N‐1\right)$$

for electrophilic attack

where $q_{k}$ (N + 1), $q_{k}$ (N), and $q_{k}$ (N − 1) are the charges at atom k on the anion, neutral, and cations species, respectively. The condensed local softness S_k_ can be easily calculated from the condensed Fukui functions $f_{k}$ and the global softness S(r) [41]. The local softness S_k_(r) is defined by:

(8) $$S_{k}^{+}\left(r\right)=S*f_{k}^{+}\;\text{for}\;\text{nucleophilic}\;\text{attack}$$

(9) $$S_{k}^{-}\left(r\right)=S*f_{k}^{-}\;\text{for}\;\text{electrophilic}\;\text{attack}$$

The local electrophilicity (ω_k_) [32] concentrated on atom k was calculated by projecting the index ω onto any reaction center k in the molecule using the Parr function f^+^_k_:

(10) $$\omega_{k}=f_{k}^{+}*\omega \;\text{for}\;\text{Nucleophilic}\;\text{attack}$$

UV-vis spectra were obtained using the TD-DFT method [42] at the same computational level. The 30 lowest electronic excitation energies ((${\Delta E}_{0n}$) and oscillator strengths ($f_{0n}$) were taken into account. The electronic spectra were simulated and analyzed using the Gaussum-3 software. The optical energy gap ${Eg}_{opt}$ can be determined by the intersection of the absorption and emission UV spectra [43].

The photostability of the chromophore plays a crucial role in determining the appropriate excitation intensity and the number of measurement cycles. These factors are of utmost importance in selecting optimal fluorescence tools, and thus, manufacturers of dyes provide data on *ε* (molar absorptivity), $\Phi_{f}$ (quantum yield), chromophore brightness, and occasionally, photostability. The FluorTools software was employed to calculate the $\Phi_{f}$ values.

#### 2.7.2 Molecular docking analysis

The primary objective of molecular docking calculations is to analyze the interactions between a ligand (organic molecule) and the amino acids present in protein receptors. In this study, **ligand 4** was screened and prepared for molecular docking with target proteins associated with antibacterial, antimicrobial, and antifungal activities. X-ray diffraction structures of resistant bacteria, including *Methicillin resistant Staphylococcus aureus* (MRSA) (PDB IDs: **1VQQ**, **1S16**, and **2 × 3F**), *E. coli MurB* (PDB ID: **2Q85**), *E. coli primase* (PDB ID: **1DDE**), *E. coli DNA GyrB* (PDB ID: **1KZN**), *Thymidyl kinase* (PDB ID: **4QGG**), *E. coli FabH* (EcFabH) in complex with Malonyl-CoA (PDB ID: **1HNJ**), and *β-Lactamase* (PDB ID: **1ONH**), were obtained from the Protein Data Bank (PDB) (https://www.rcsb.org/). Docking calculations were performed using the EZACDD Smina [44] package program, and the resulting complexes were visualized using Discovery Studio software 2021 client program. Prior to docking, water molecules and ligands were removed using Pymol [45], and automatic cavity detection was conducted using *fpocket3* [46]. The docked complexes were evaluated based on the bonding interaction patterns and minimum binding affinity values.

## 3 Results and discussion

### 3.1 Theoretical study

#### 3.1.1 Global reactivity

The reaction mechanism between the reactants: 4-nitrobenzaldehyde**R**_**1**_, 1-hydroxy-2-naphathadehyde **R**_**2**_and benzene-1.4-diamine **R**_**3**_ is sketched in Scheme 1.

For a reaction between A and B molecules, the global reactivity analysis allows us to determine the nucleophilic and electrophilic reagents. The assignment was based on computing two energy parameters ΔE1 and ΔE2 and both equations are expressed as follows:

(11) $$\Delta E1=E_{\text{LUMO}\;\left(A\right)}\;–\;E_{\text{HOMO}\;\left(B\right)}$$

(12) $$\Delta E2=E_{\text{LUMO}\;\left(B\right)}\;–\;E_{\text{HOMO}\;\left(A\right)}$$

**Fig. 1:**
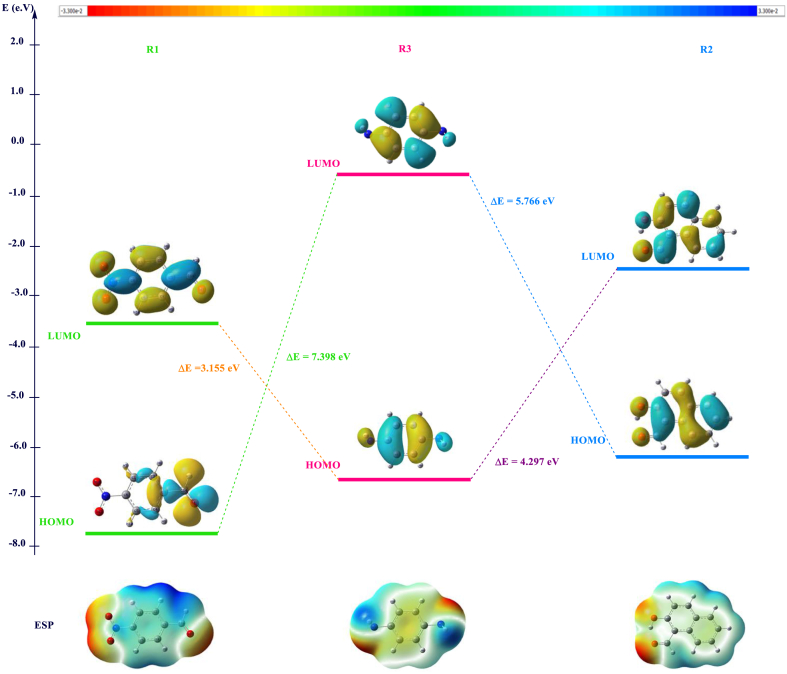
Computed MESP, HOMO, LUMO and energy gap values in (eV) of **R**_**1**_**-R**_**3**_ and **R**_**2**_**-R**_**3**_ reacting systems at DFT/B3LYP/6-31G+(d) level.

If ΔE1 < ΔE2; we assign A as acceptor and B as the donor and consequently, the electrophilic and the nucleophilic reagent, respectively. The computed Ɛ_LUMO,_ Ɛ_HOMO,_ the mapped electrostatic potential (MESP) surfaces of the reactants **R**_**1**_, **R**_**2**_ and **R**_**3**_ and the combined energy gap (ΔE) values are illustrated in Fig. 1 and in Table 1.

**Table 1:** Computed global and local descriptors of the **R**_**1**_**, R**_**2**_and **R**_**3**_ at the B3LYP/6-31+G(d) level.

	E_HOMO_(eV)	E_LUMO_(eV)	*η* (eV)	*μ* (eV)	*ω* (eV)	*S* (eV)	$q_{k}$ (N)	$q_{k}$ (N+1)	$f_{k}^{+}$	$S_{k}^{+}$	ω_k_ (eV)	V_S,max_ (a.u)
R_1_ (Carbonyl)	−7.938	−3.564	4.374	−5.751	3.780	0.114	−0.396	−0.323	0.073	0.00832	0.279	0.027
R_2_ (Carbonyl)	−6.298	−2.414	3.884	−4.356	2.442	0.128	−0.381	−0.329	0.052	0.00665	0.127	0.007
R_3_	−6.723	−0.524	6.199	−3.623	1.059	0.080						

According to the calculated energy gap (ΔE) values in Fig. 1, 1-hydroxy-2-naphathadehyde **R**_**2**_ is the donor (nucleophilic) molecule while 4-nitrobenzaldehyde **R**_**1**_, and benzene-1.4-diamine **R**_**3**_ are the acceptors (electrophiles)**.** In the case of R1, The HOMO is localized in aromatic ring and carboxylic acid, while the lumo is displaced to the nitrogen dioxide.

The molecular electrostatic potential at a given point around a molecule is defined in terms of the total charge distribution of the molecule and relates to dipole moments. It provides a method to understand the electron density, which is useful for determining the electrophilic and nucleophilic reactivity [[47], [48], [49]]. In Fig. 1, the negative charges and, consequently, the nucleophilic region are colored in red, whereas the positive region, which is related to electrophilic sites, is colored in blue. The light blue and yellow indicate a slightly electron-deficient region and a slightly electron-rich region, respectively [[50], [51], [52], [53], [54]]**.**

In the case of the reactants **R**_**1**_ and **R**_**2**_, carbonyl group is the most electrophilic site. For **R**_**3**_,the amine groups are the most nucleophilic sites. Furthermore, the aromatic electronic cloud becomes more reactive when comparing the color evolution in the case of the R3 product with both R1 and R2 reagents.

#### 3.1.2 Local reactivity

Table 1 summarizes the global and local descriptors of all reactants. The obtained results indicated that the global hardness increases for compounds R_**1**–**3**_ as follows: **R**_**3**_ > **R**_**1**_ > **R**_**2**_ and the chemical reactivity decreases in the opposite order: **R**_**3**_ < **R**_**2**_ < **R**_**1**_. Reactants with greater values of chemical potential are more reactive than those with small electronic chemical potentials ones [55]. Electrophilicity (ω) is an important descriptor of reactivity that allows the quantitative classification of the global electrophilic nature of a molecule on a relative scale. The classification of organic molecules as strong electrophiles The electrophilicity of organic molecules can be classified as follows: strong electrophiles if ω > 1.5 eV, moderate electrophiles if 0.8 <ω < 1.5 eV, and marginal electrophiles if ω < 0.8 eV [43]**.** Regarding the data in Table 1, the ω values of **R**_**1**_,**R**_**2**_ and **R**_**3**_ are equal to 3.780 eV, 2.442 eV and 1.059 eV, respectively, which shows clearly, that the component **R**_**1**_ can be considered a strong electrophile and the most reactive.

As a relevant result, we have found that R1 carbonyl is more reactive than R2, and this is in good agreement with the global reactivity study. Also, the analysis of the maximum potential $V_{S,\max}$ values confirms this result.

#### 3.1.3 Transition state (TS) and intrinsic reaction coordinate (IRC) calculations

The aim of this section is to investigate the kinetic and thermodynamic profiles of the studied reactions. The kinetic reaction exhibits lower activation energy $E_{a}$ value and the thermodynamic one possesses the highest free energy of reaction $\Delta_{r}E$ absolute value. Both parameters are deduced from the computed IRC plots and they can be expressed as follows:

(13) $$E_{a}=E\left(\text{transition}\;\text{state}\right)\;–\;E\left(\text{reactant}\right)$$

(14) $$\left|\Delta_{r}E\right|=\left|E\left(\text{product}\right)\;–\;E\left(\text{reactant}\right)\right|$$

**Fig. 2:**
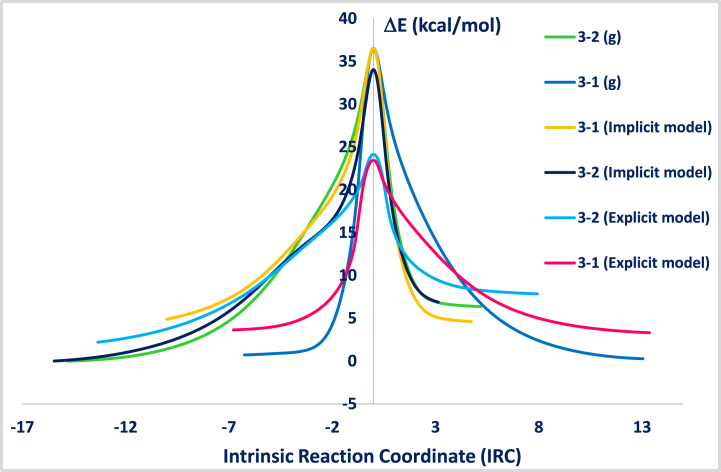
Computed IRC plots of the reaction between benzene-1,4-diamine **R**_**3**_ with 4-nitrobenzaldehyde **R**_**1**_(**3**–**1**) and benzene-1,4-diamine **R**_**3**_ with 1-hydroxy-2-naphathadehyde **R**_**2**_ (**3**–**2**) in gas phase and in ethanol solvent (implicit and explicit model) at 6-31G+(d) level.

The calculations were carried out in gas phase and in solvatation model (implicit and explicit models) and the IRC plots are illustrated in Fig. 2. $E_{a}$ and $\Delta_{r}E$ values are shown in Table 2.

**Table 2:** Computed activation energy (E_a_) and free energy of reaction (Δ_r_E) values in (kcal/mol) of the reactions **(3**–**1)** and **(3**–**2)** at B3LYP/6-31+G(d) level as a function of the solvatation model.

*Reaction*	Vacuum		Explicit model		Implicit model	
	$E_{a}$	$\Delta_{r}E$	$E_{a}$	$\Delta_{r}E$	$E_{a}$	$\Delta_{r}E$
***3–1***	36.31	−0.45	20.16	−0.34	31.36	−0.26
***3–2***	36.38	6.37	21.94	5.65	34.03	6.88

In the case of the reaction 3-1 (between R3 and R1) (3-1), the explicit model yielded the lowest activation energy value (E_a_ = 20.16 kcal/mol). The explicit model, which shows the stability of the intended product, was found to be quite appropriate for the reaction under study.

#### 3.1.4 Mechanism study proposition

**Fig. 3:**
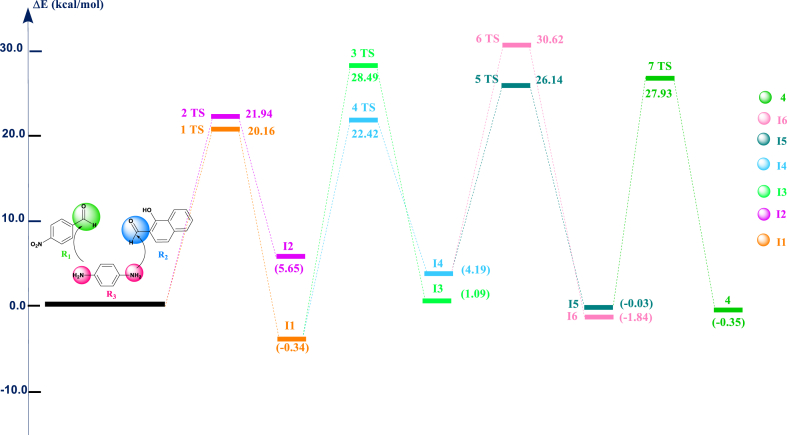
Computed energy profile of the component **4** at B3LYP/6-31+G(d) level in ethanol solvent (Explicit model).

**Scheme 2:**
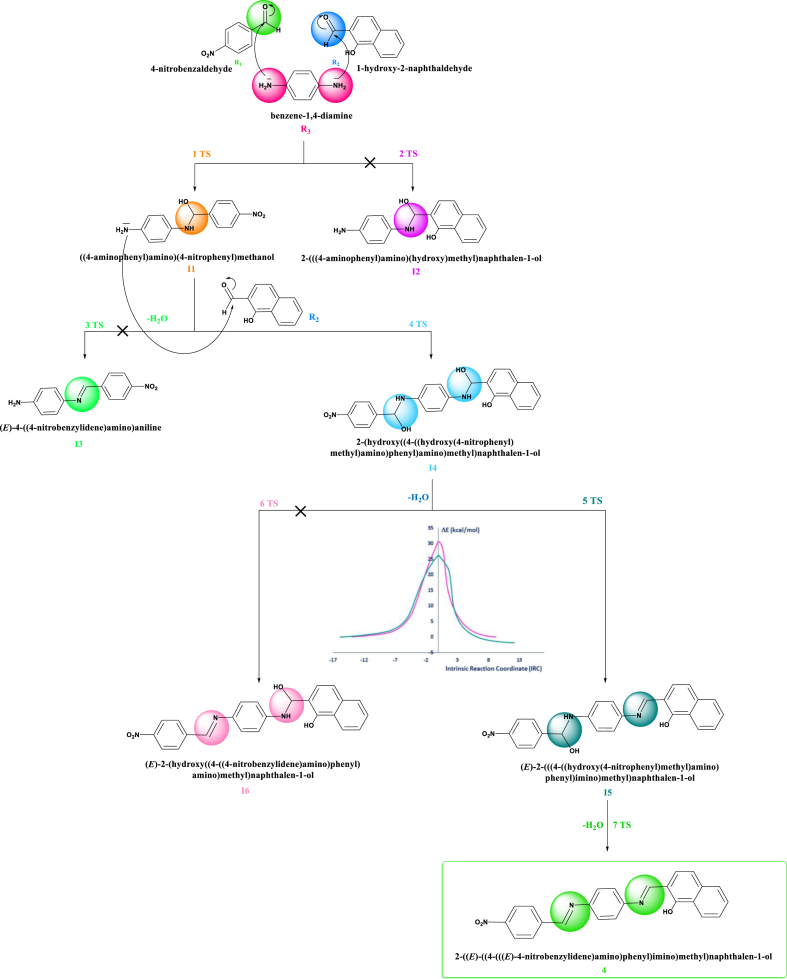
Proposed mechanism and computed IRC plots at B3LYP/6-31+G(d) level for synthesis of component **4** in ethanol using explicit model.

The component **4, 2-((E)-(4-((E)-4**-nitrobenzylidene)amino)phenyl)imino)methyl) naphthalenol, was synthesized by reacting three components, R_1_, R_2_, and R_3_, of a *one-pot* condensation reaction in ethanol heated under reflux. The reaction mechanism was studied to explain the efficient chemical transformation to product **4** in a single operation. Fig. 3 and Scheme 2 provided a graphical representation of the calculated reaction pathways.

The mechanism of the reaction, where benzene-1,4-diamine **R3** directly adds to 1-hydroxy-2-naphthaladehyde **R2**, was calculated. This reaction mechanism proved to be highly unlikely. Then, we have searched for another reaction pathway. Indeed, an alternative pathway was found, which is considerably more probable. The reactant of the benzene-1,4-diamine **R**_**3**_ containing two nucleophilic (-NH_2_ group), this nucleophile-initiated to attack the most electrophilic center (the carbonyl group of the reactant **R**_**1**_) led to the intermediate **I1** with a lowest kinetic barrier (E_a_ = 20.16 kcal/mol). The second pathway favors the attack of amino group of the intermediate **I1** of the electrophilic center (aldehyde function introduced on the 1-hydroxy-2-naphathadehyde **R**_**2**_) with activation energy value (E_a_ = 22.42 kcal/mol) form the intermediate **I4**.

According to the experiment data, the reaction mixture was heated under reflux that is facilitate the elimination of two water molecules. The study the removal of two water molecules was studied, which explained that the first elimination of water molecule started from the fragment –NH–CH(OH) – related naphthalene-1-ol fragment led to intermediate **I5** with activation energy value equal to 26.14 kcal/mol. The elimination the second water molecule generates the formation of the product **4**.

The reaction mechanism of all pathways showed in details in Fig. 3 and in Scheme 2.

### 3.2 TD-DFT absorption UV spectra analysis

**Fig. 4:**
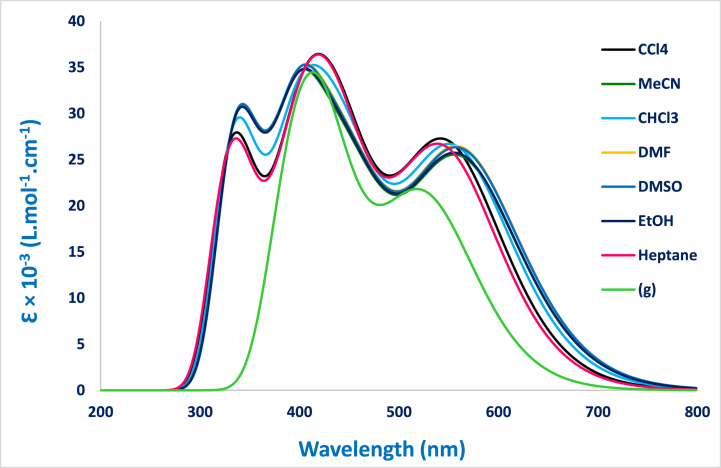
Computed UV-abs spectra at TD-DFT B3LYP/6-31+G(d) level as a function of different solvents.

**Fig. 5:**
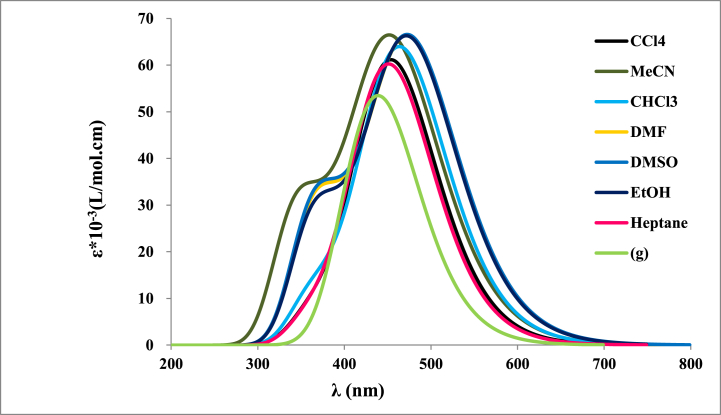
Computed UV-emission spectra at TD-DFT B3LYP/6-31+G(d) level as a function of several solvents.

The theoretical absorption spectra for dimine product **4** were sketched in Fig. 4 and discussed. The computed emission spectra of the studied dye in different solvent are illustrated in Fig. 5.

The UV absorption spectra showed generally three bands, two in the 350–450 nm and the third one appears in 500–570 nm. Those UV band spectra can be assigned to the π-π* electronic transition. The determined optical characteristics issued from the UV absorption spectra are summarized in Table 3. The highest oscillator strength values are observed in the case of DMF and DMSO. A red shift by 20 nm was observed when using a polar solvent. The analysis of the data in Table 4 shows that the optical energy gap ${Eg}_{opt}$ values decrease as a function of the polarity of the solvents. The lowest ${Eg}_{opt}$ value (2.18 eV) was observed in the case of acetonitrile.

**Table 3:** Computed absorption wavelength $\lambda_{TD-DFT}$ in (nm), vertical transition energy ${\Delta E}_{0-0}$ in (eV), molecular orbitals involved in the transitions (major contributions) and oscillator strength $f_{0n}$ values of dimine compound **4** at TD-DFT/B3LYP/6-31+G(d).

Solvant	$\lambda_{TD-DFT}$	${\Delta E}_{0-0}$	MOs	$f_{0n}$
**Gas phase**	413	2.36	H→L(+1) (94%)	0.81
		2.90	H(-1)→L (95%)	
	519	3.17	H→L (99%)	0.35
**Tetrachloromethane**	356	3.83	H(-5)→L (58%)	0.55
		3.58	H(-1)→L(+1) (80%)	
	412	3.11	H→L(+1) (95%)	0.88
		3.79	H(-1)→L (94%)	
	548	2.26	H→L (97%)	0.48
**Chloroform**	352	3.79	H(-5)→L (54%)	0.56
		3.57	H(-1)→L(+1) (83%)	
	401	3.10	H→L(+1) (95%)	0.89
		2.75	H(-1)→L (95%)	
	556	2.23	H→L (98%)	0.47
**Acetonitrile**	352	3.77	H→L(+2) (55%)	0.46
		3.56	H(-1)→L(+1) (68%)	
	390	3.11	H→L(+1) (95%)	0.90
		3.72	H(-1)→L (96%)	
	562	2.20	H→L (98%)	0.45
**Dimethylformamide**	351	3.77	H→L(+2) (57%)	0.47
		3.56	H(-1)→L(+1) (70%)	
	392	3.10	H→L(+1) (95%)	0.92
		2.72	H(-1)→L (95%)	
	563	2.20	H→L (98%)	0.47
**Dimethylsulfoxide**	352	3.77	H→L(+2) (58%)	0.47
		3.56	H(-1)→L(+1) (69%)	
	392	3.10	H→L(+1) (95%)	0.93
		2.72	H(-1)→L (96%)	
	563	2.20	H→L (98%)	0.47
**Ethanol**	351	3.71	H→L(+2) (53%)	0.48
		3.53	H(-1)→L(+1) (70%)	
	391	3.11	H→L(+1) (95%)	0.89
		2.73	H(-1)→L (96%)	
	562	2.20	H→L (98%)	0.46
**Heptane**	339	3.84	H(-5)→L(+2) (57%)	0.54
		3.59	H(-1)→L(+1) (70%); H(-2)→L (45%)	
	417	3.11	H→L(+1) (95%)	0.87
		2.80	H(-1)→L (94%)	
	545	2.27	H→L (98%)	0.47

**Table 4:** Computed intersection $\lambda_{\mathrm{int}}$ and excitation $\lambda_{exc}$ wavelengths in (nm), optical energy gap ${Eg}_{opt}$ in (eV), and fluoresence quantum yield $\Phi_{f}$ of dimine product **4** at TD-DFT/B3LYP/6-31+G(d).

	$\lambda_{\mathrm{int}}$	${Eg}_{opt}$	$\lambda_{exc}$	$\Phi_{f}$
**Gas phase**	511	2.43	400	0.13
**Tetrachloromethane**	529	2.34		0.15
**Chloroform**	548	2.26		0.17
**Acetonitrile**	568	2.18		0.18
**Dimethylformamide**	566	2.19		0.18
**Dimethylsulfoxide**	567	2.19		0.18
**Ethanol**	566	2.19		0.18
**Heptane**	526	2.36		0.15

We have also obtained the highest quantum yield fluorescence yield $\Phi_{f}$ in the case of the polar solvents. We can conclude that it’s recommended the reaction medium should include solvents (Acetonitrile, DMF, DMSO and ethanol).

#### 3.2.1 TD-DFT fluorescence UV spectra analysis

The computed emission spectra of the studied dye in different solvent are indicated in Fig. 5.

### 3.3 Measured absorption and emission UV spectra

#### 3.3.1 Effect of different media

**Fig. 6:**
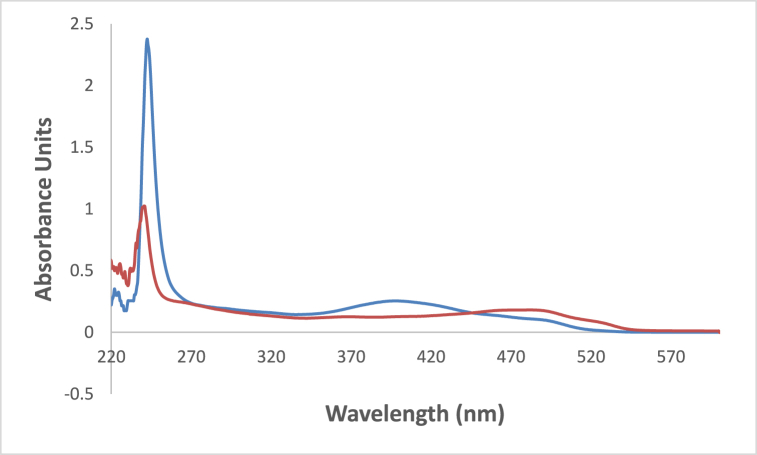
Absorption spectra of 1.2 × 10^−5^ M 4NMN in CHCl_3_ and Glycerol solvents.

**Fig. 7:**
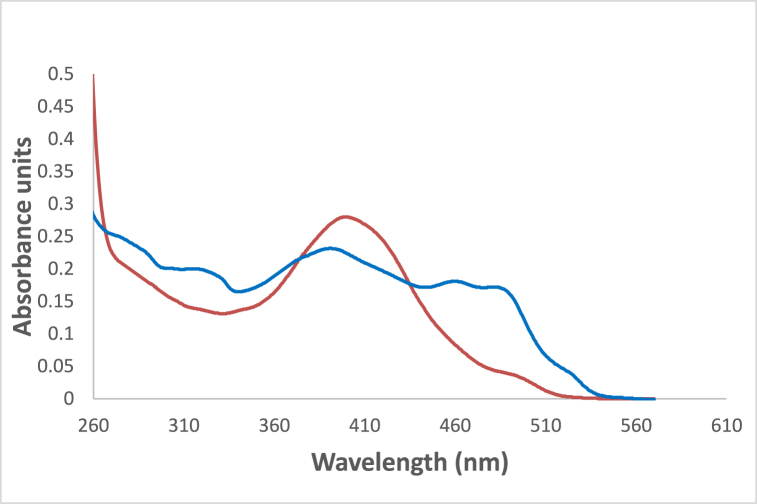
Absorption spectra of 1.2 × 10^−5^ M H-NO_2_ in  ethanol and  CCl_4_.

In Fig. 6 the studied dye In chloroform solvent, 4NMN shows distinct and high absorbance at 243 nm due to the spin−allowed S_0_ → S_2_ transition. The appearance of absorption bands at lower energy for the studied dye at lower energy 396 and 483 nm, in chloroform and glycerol solvents respectively is attributed to a spin−allowed S_0_ → S_1_ transition. The longer wavelength shift observed in glycerol absorbance at the S_0_ → S_1_ peak than that in CHCl3 solvent is credited to the hydrogen-bonding interaction between the NO_2_ lone pair of electrons and the HBD solvent molecules [56]. This behavior is supported by Fig. 7, if comparing with the effect of ethanol and carbon tetrachloride solvents.

**Fig. 8:**
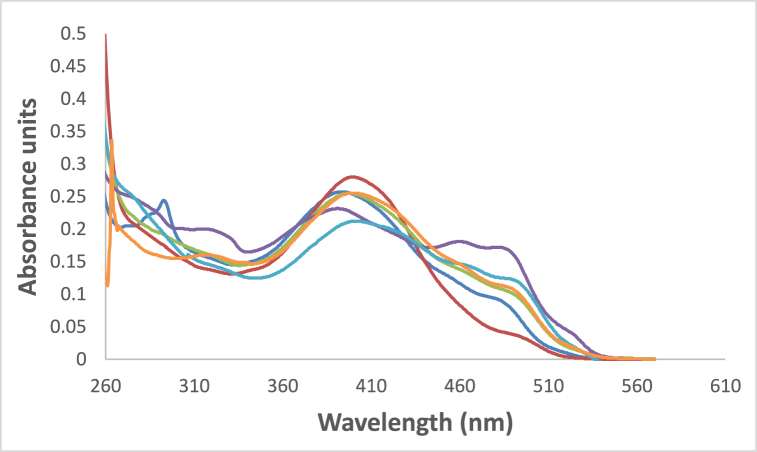
Absorption spectra of 1.2 × 10^−3^ M 4NMN in CH_3_CN,  CHCl_3_, CCl_4_, ethanol, DMSO and  DMF.

**Fig. 9:**
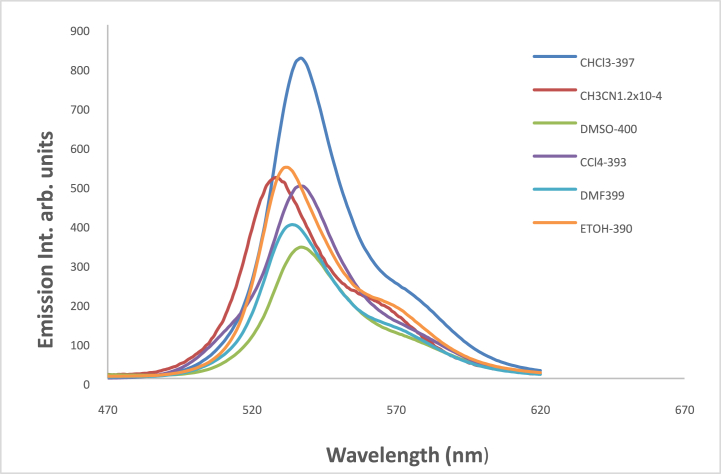
Emission spectra of 1.2 × 10^−5^ M 4NMN inheptane, CCl_4_, CHCl_3_,  ethanol, DMSO, DMF and CH_3_CN.

**Fig. 10:**
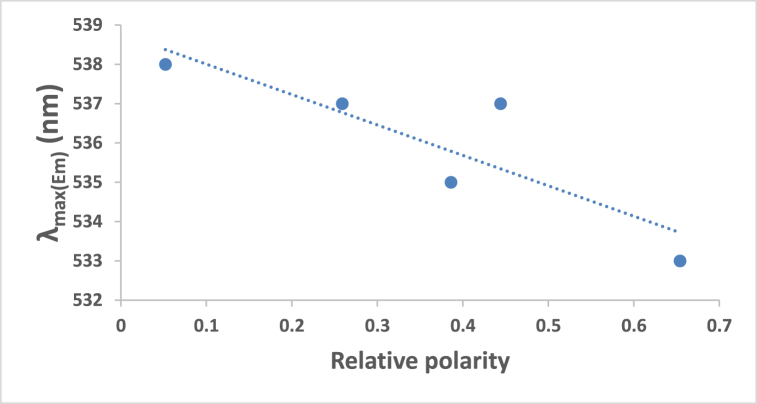
Relative polarity effect on the emission maxima wavelength (*λ*_em)_max of 1.2 × 10^−5^ M 4NMN dye.

**Fig. 11:**
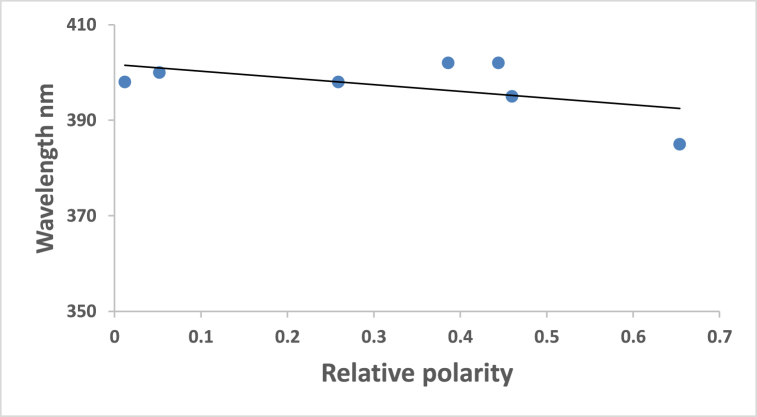
Effect of relative polarity on the absorption maxima wavelength (*λ*_abs_ max) of 1.2 × 10^−5^ M 4NMN.

At ambient temperature and a concentration of = 1.2 × l0^−5^ mol dm^−3^, the UV/Vis absorption, emission, and excitation spectra of the 4NMN dye were obtained in several solvents with different polarities. Fig. 8, Fig. 9, Fig. 10, Fig. 11 demonstrate how the solvent polarity affects where the electronic absorption and emission maxima are located. As the solvent polarity of the medium increases, the hypsochromic band shifts in the electronic absorption, and emission spectra of 4NMN are expected and reflect a decrease in the dye molecule's ground-state dipole moment upon excitation and an increase in its ground-state dipole moment as a result of solvent polarization [[57], [58], [59]]**.** Variation of fluorescence intensity of 4NMN in different solvents indicate also the effect of solvents on the Fluorescence efficacy and fluorescence quantum yield (**φ**_**f**_) as shown in Fig. 9 and Table 1. The UV/Vis spectral measurements are summarized in Table 5. The experimental UV spectra shapes are close to those computed theoretically.

**Table 5:** Spectral characteristics and photo physical parameters of Coumarin derivative (4NMN) in different solvents.

Solvents	E_T_(30) Kcal/mol	Relative polarity	Δf	*λ*_em max_(nm)	*λ*_abs__max_(nm)	*ε*_max__So-S1__transition_ dm^3^.mol^−1^ cm^−1^	φ_f_
**CCl**_**4**_	32.4	0.052	0.115	538	400	23,333	0.18
**CHCl**_**3**_	39.1	0.259	0.251	537	398	21,250	0.3
**DMF**	43.8	0.386	0.377	535	402	21,250	0.14
**DMSO**	38.4	0.444	0.373	537	402	17,666	0.12
**CH3CN**	45.6	0.460	0.386	529	395	21,166	0.19
**EtOH**	51.9	0.654	0.379	533	385	19,583	0.2
**Glycerol**	57.0	0.812	0.32		481	15,166	
**Heptane**		0.012		533	398		

**Fig. 12:**
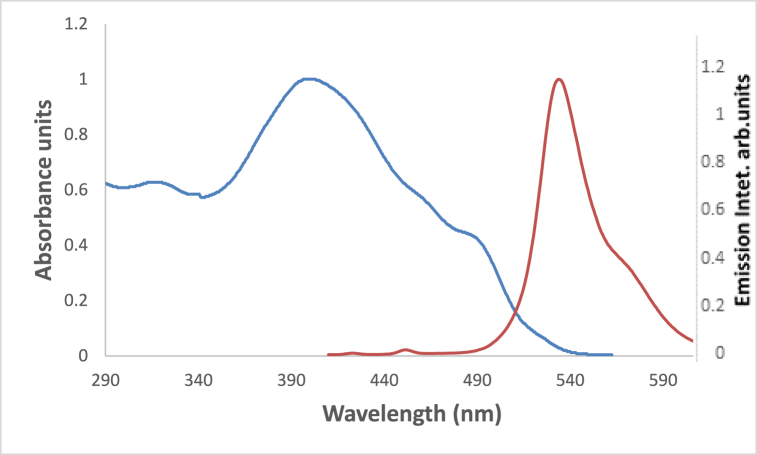
Normalized absorption, fluorescence  of 1. × 10^−5^ M 4NMN in DMF (*λ*_ex_ 399 nm).

Due to the initial excited state's vibrational relaxation to the first electronic excited state's lowest vibrational state, a substantial stock shift of approximately 135 nm was observed for 1.2 × 10^−3^ M of 4MNM in DMF. Emission occurs as a result of returning to a variety of vibrational levels in the ground state, causing a redshifted emission as shown in Fig. 12. Another explanation for the redshift in the emission could be the development of a highly polar excited state that immediately follows the excitation relaxes the solvent cage around it.

**Fig. 13:**
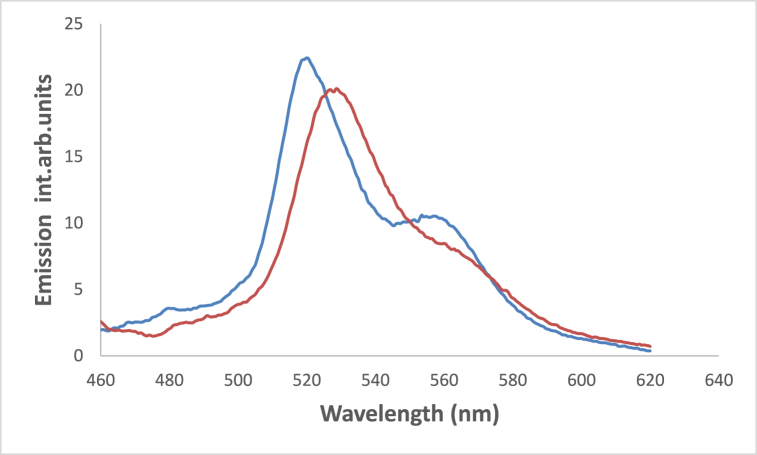
Emission spectra of 1.2 × 10^−4^ M 4NMN in CH_3_CN fresh, and  acidic, λex-449nm.

In acidic media, a structured fluorescence spectra is formed where a new emission band developed at 558 nm together with a hypsochromic shift in the position of the emission maximum from 528 nm in fresh CH_3_CN to 520 nm in acidic medium (Fig. 13). This shift results from the protonation of olefinic nitrogens with subsequent development of the spectral patterns [60]**.**

**Fig. 14:**
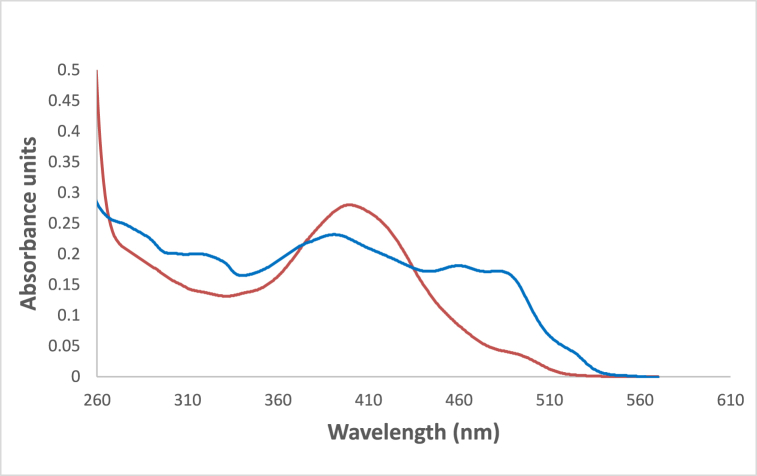
Absorption spectra of 1.2 × 10^−5^ M 4MN in  ethanol and 3 CCl_4_.

**Fig. 15:**
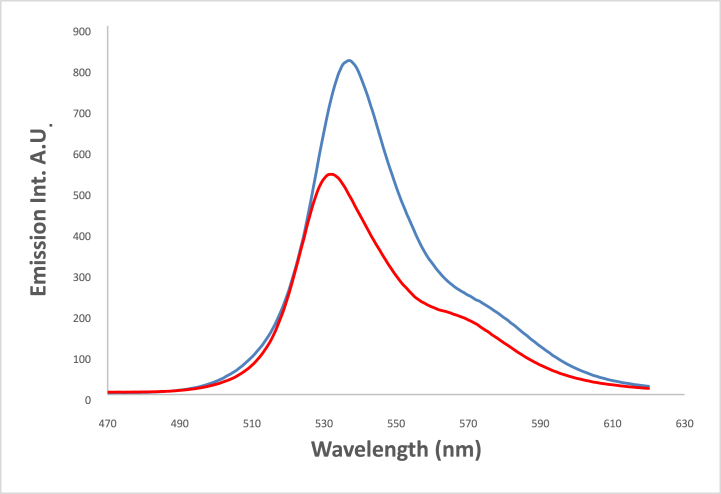
Fluorescence spectra of 1.2 × 10^−3^ M 4NMN in CHCl_3_, and  ethanol, λex. = 396 nm.

In nonpolar solvents (CHCl_3_, and heptane) as shown in Fig. 14, the absorption spectra of 4NMN exhibit more structure when compared to that in ethanol solvent (polar solvent). This may be caused by a specific solvent-solute interaction (hydrogen bonding with the dye molecules). The blue shift in the emission spectrum of the dye in chloroform solvent compared to ethanol or glycerol is the result of the absence of the effect of polarity and the effect of hydrogen bonding combined (see Fig. 15).

**Fig. 16:**
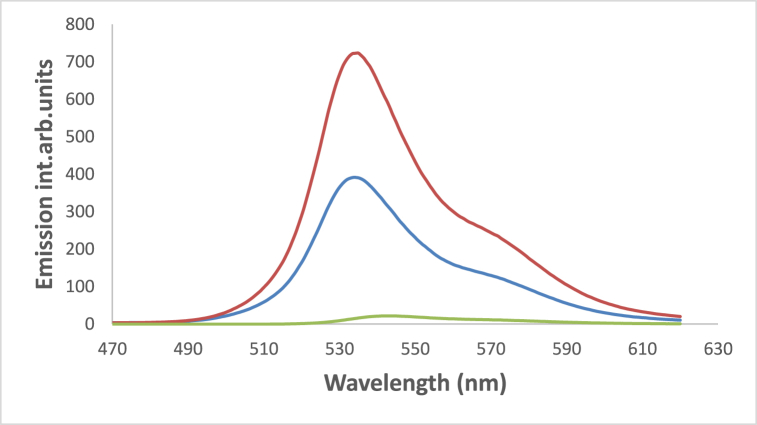
Fluorescence intensities of 4NMN in DMF at 1.2 × 10^−3^ M , 3.6 × 10^−3^ M and 2 × 10^−1^ M, λex 396 nm.

Fig. 16 shows that the emission intensity of the 4NMN in DMF increases with increasing concentration from 1.2 × 10^−3^ to 3.6 × 10^−3^. Excessive increase (fifty time) in dye concentration (from 3.6 × 10^−3^ 2 × 10^−3^ resulted in decreasing emission intensity about 30 times with increased concentration, due to the inner filter effect. A small red shift (8 nm) in emission maximum wavelength.

**Fig. 17:**
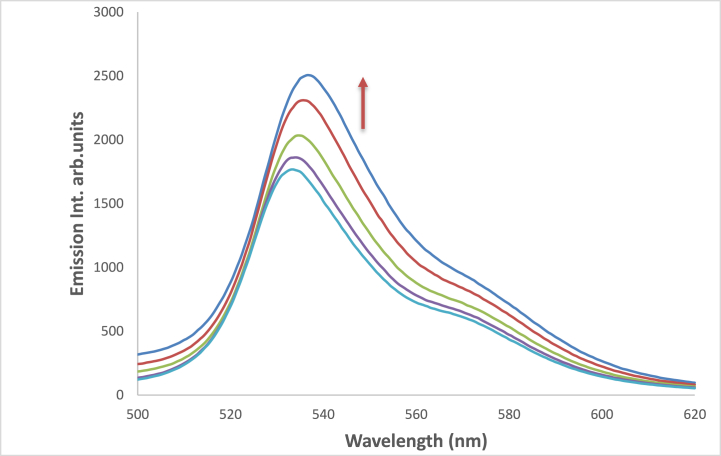
Fluorescence spectra of 1.2 × 10^−5^ M 4NMN in ETOH/Glycerol mixture (*λ*_em_ max = 533 nm) increasing of emission intensity at increasing glycerol concentration percentage, 0, 20, 40, 80 and 100%, λex = 395 nm.

**Fig. 18:**
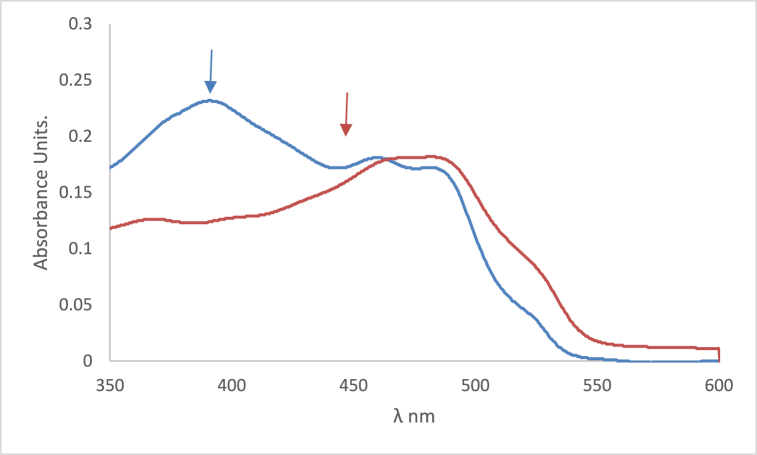
Absorption spectra of 1.2 × 10^−3^ M 4NMN in ethanol , glycerol.

Due to the involvement of viscosity of the media in producing large bathochromic shifts of absorption maximum wavelength, which is explained by the role of the medium cage in maintaining molecule planarity in electronically excited states, and a subsequent drop in potential energy due to extended conjugation [[61], [62], [63]]**.** This effect plays a crucial role in enhancing both bathochromic shifts as well as fluorescence efficiencies where a successive increase in emission intensity was increased by increasing glycerol percent in the solution as shown in Fig. 17. For two protic solvents of similar polarities, e.g., ethanol and glycerol, the maximum of the absorption peaks were shifted from 392 to 488 nm see Fig. 18.

#### 3.3.2 Quenching by metal ions

**Fig. 19:**
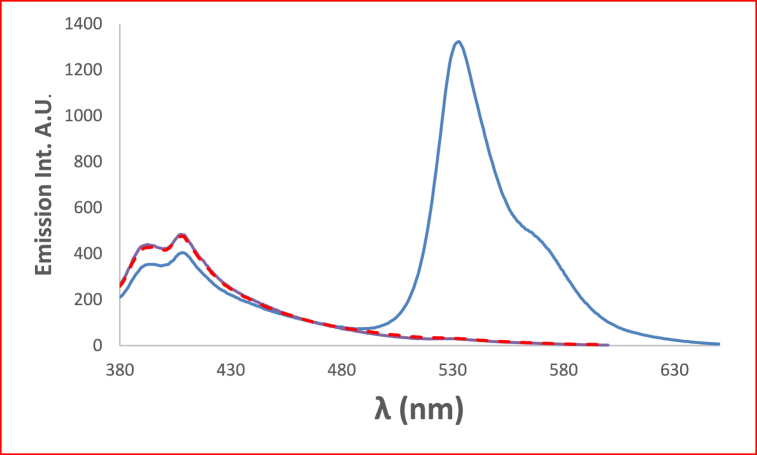
Fluorescence spectra of 1.2 × 10^−3^ M 4NMN in ethanol fresh Emission intensity decreases with the addition of similar concentrations of different metal Ions of 25 μM, Fresh  Cr^2+^, and Cu^2+^, inset is acrossectional of emission intensies at 530 nm, λex = 396 nm.

**Fig. 20:**
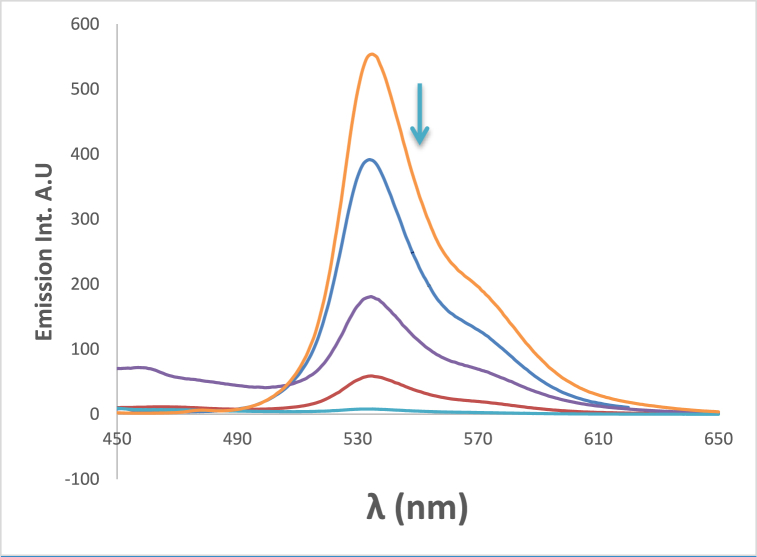
Fluorescence spectra of 1.2 × 10^−3^ M of 4NMN in DMF; decrease in emission intensity at increasing [Cr^3+^]: 0, 4, 6, 10, 12 μM, *λ*_ex_ = 396 nm.

The addition of chromium and copper ions causes fluorescence quenching due to the heavy atom effect and paramagnetic nature of Cu^2+^and Cr^3+^ as shown in Fig. 19, Fig. 20. A gradual decrease in the emission intensities of the dye at 530 nm with successive addition of Cu^2+^ in the form of copper sulfate pentahydrate, accompanied by a slight bathochromic shift of ca. 3 nm in ethanol. This quenching of emission intensities by both metals is evidence of the dye's efficacy in identifying small amounts of these two metals [64]. Also the decrease in H-bonding due to metal coordination with Schiff bases can contribute to fluorescence quenching [[65], [66], [67], [68], [69]].

### 3.4 Biological evaluation

The compounds containing the azomethine group (–N=C–) are important compounds in biological studies, as they are an active group in coordination and bonding. When studying this compound on bacteria *Salmonella enterica* serovar typhi and fungi type *Candida Albicans.*

It showed a high activity towards. fungi but it did not show any activity toward bacteria The reason for that may be due to the large structural shape of this compound (Table 6). While it was studied on some cancer cells of type HepG-2, SKOV3, PC-3, and HeLa. The compound showed high effectiveness against some cells, while its effect was not high on others see Table 7.

**Table 6:** The IC_50_ and CC_50_values (μg/mL) of the tested ligand against different human cell lines.

No.	Compound	IC_50_ (μg/mL)			CC_50_ (μg/mL)
		HepG-2	A-549	PC-3	WI-38
1	**[ C**_**24**_**H**_**17**_**N**_**3**_**O**_**3**_**]**	87.6 ± 4.06	60.9 ± 2.85	111.1 ± 3.29	172.51 ± 5.17

**Table 7:** Antimicrobial activities of different levels of the ligand and its metal complexes against *S. enterica* ser. Typhi bacteria and *C. albicans* fungi.

No.	Compound	*S. enterica ser. Typhi* (mg/mL)			*C. albicans* (mg/mL)		
		30	20	10	30	20	10
–	**DMSO**	0	0	0	0	0	0
**1**	**[HL][ C**_**24**_**H**_**17**_**N**_**3**_**O**_**3**_**]**	0	0	0	11	9	7
–	**Gentamycin**	–	–	17	–	–	–
–	**Clotrimazole**	–	–	–	–	–	21

Note: Inhibition in mm.

#### 3.4.1 Antimicrobial activities of the para-diamine derivative against the growth of *S. enterica* ser. Typhi and *C. Albicans*

The data showed represent antifungal activities of the studied dye at different volumes against the growth of *C. albicans*. The dye had high activities at 30 mg/mL, while it had intermediate activities at 20 mg/mL. The result showed no antibacterial activities of the compound at 30, 20 and 10 mg/mL against the growth of *S. enterica* ser. Typhi.

### 3.5 Molecular docking analysis

#### 3.5.1 Antibacterial

Based on the clear zones and MIC values, we investigated the probable mechanism of action of the designed compound by performing docking studies to several antibacterial targets, including *E. coli* Primase, *E. coli* DNA GyraseB, *S. aureus* Thymidylate kinase, β-Lactamase, and *E. coli* FabH. The docking studies revealed that the binding free energy to the different receptors was higher than that to Thymidyl kinase *E. coli* MurB enzyme and β-Lactamase. The most active compound, Compound 4, showed the most favorable hydrogen bond interactions with *E. coli* MurB enzyme. The results of the docking exploration showed that the selected ligand, Compound 4, had binding affinities ranging from −8.0 to −10.0 kcal/mol.

Hydrogen bonds were observed between the hydroxyl substituent of the benzene ring of the compound and the residues MET268, ASP169, ASP73, and ASP316 (at distances of 2.66 Å, 2.18 Å, 4.39 Å, and 2.05 Å, respectively). Another hydrogen bond interaction between the oxygen of the NO_2_ fragment and the residues ASP107, ASN51, ASN152, GLN120, GLY49, and SER50 (at distances of 3.32 Å, 3.33 Å, 3.19 Å, 3.01 Å, 3.22 Å, and 2.85 Å, respectively) was found.

The phenyl rings interacted via hydrogen bond interactions with the residues THR16, SER116, and ARG315 (at distances of 3.76 Å, 3.68 Å, and 3.11 Å, respectively). Furthermore, the imine fragment (-C=N) contributed to the antibacterial activity of the compound by establishing a hydrogen bond with the residues THR16, THR91, ARG76, ASN193, and ALA86 (at distances of 3.33 Å, 3.91 Å, 4.80 Å, 3.20 Å, and 3.69 Å, respectively).

These interactions stabilized the complex compound-enzyme and played a crucial role in the increased inhibitory action of Compound 4 (see Table 8 for more details).

**Table 8:**
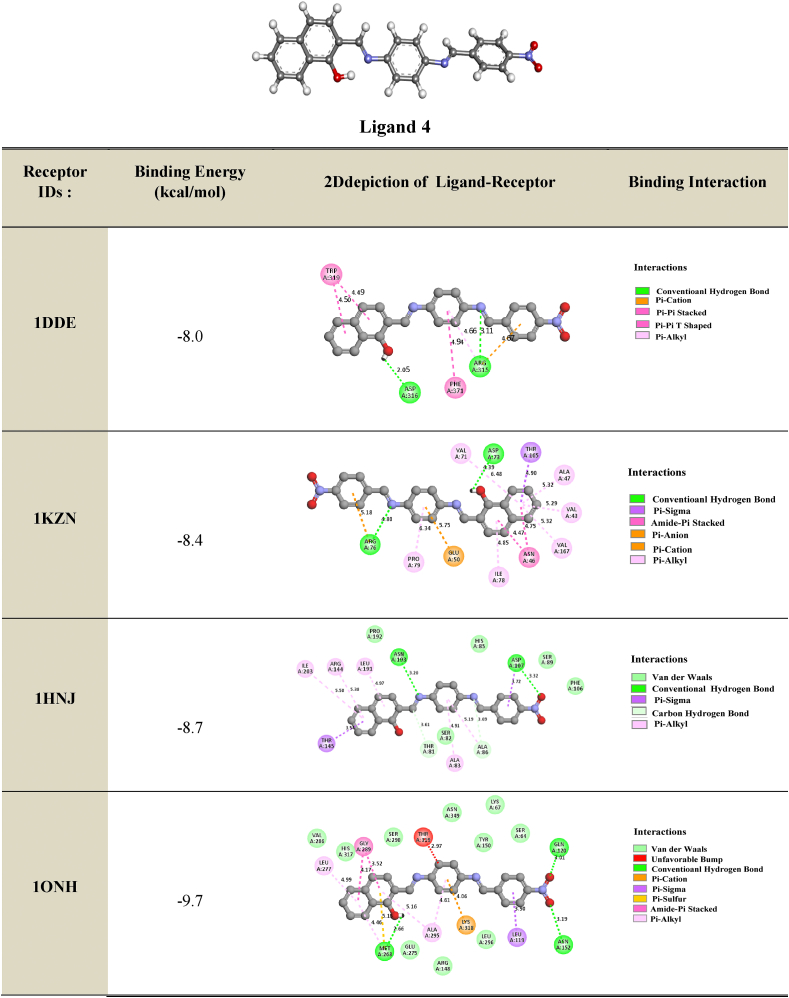

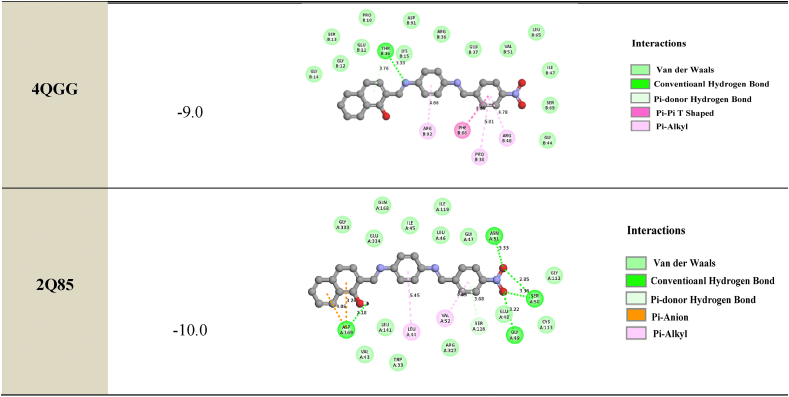
2D depiction of docking of ligand **4** with different proteins (Binding Energy in kcal/mol).

#### 3.5.2 Antifungal

We investigated the anti-MRSA activity of ligand 4 by performing molecular docking studies to different antifungal targets. MRSA is a major cause of nosocomial infections and its increasing prevalence has been observed in the last decade. The treatment of MRSA infections is difficult due to the restricted spectra of efficient antibiotics. Our docking results showed that the binding energies of the best-docked compound ranged between −7.8 kcal/mol and −9.7 kcal/mol (Table 9). We found that the azote of the imine group interacts with the residues SER240 and TRP2357, which is the hydrogen bond distance *δ* (*δ* = 2.95 Å and 3.57 Å, respectively). Another hydrogen bond interaction was observed between the hydroxyl substituent of the benzene ring of the compound and the residues ALA2353 (*δ* = 3.30 Å) and VAL2349 (2.10 Å). Furthermore, the oxygen of the NO_2_ interacts via a hydrogen bond with the residues ARG241 (3.03 Å) and ARG151 (3.05 Å). An electrostatic bond (Amide Pi stacked, Pi-pi T shaped, Pi cation, Pi anion, Pi sulfur and Pi sigma) was also found between the benzene ring with the residues ASP2352 (4.16 Å), ALA2353 (3.90 Å), TRP2357 (4.54 Å), VAL15 (3.53 Å), TYR373 (5.04 Å), and MET141 (*δ* = 2.39 Å). The benzene ring interacts hydrophobically with the residues ARG241, LYS215, ALA168, LYS16, LYS19, VAL15, ALA168, and ILE2239. These interactions stabilize the complex compound enzyme and play a crucial role in the increased inhibitory action of the component 4. 2D-depictions showed some hydrogen bond interactions in green color and Pi-alkyl interactions in party pink color. An electrostatic bond such as Pi-sigma interaction was shown in amethyst color, Pi-pi-T shaped, Pi-pi stacked and amide pi-stacked interactions were revealed in fuchsia color and Pi-cation, Pi-anion and Pi-sulfur were exhibited in marigold color (Table 8, Table 9). In the culmination of our investigation into the biological activities of the compound, the alignment between experimental results and molecular docking analyses underscores the validity of our predictions regarding its mode of action. The compound's observed high antifungal potency against Candida Albicans and its selective impact on specific cancer cells find substantiation in the molecular interactions identified through docking studies. Notably, the hydrogen bond interactions between Compound 4 and key residues of antibacterial and antifungal targets provide crucial insights into its dual antimicrobial potential. This convergence of experimental evidence and computational predictions not only enhances our understanding of the compound's biological activities but also serves as a robust foundation for future endeavors in harnessing its therapeutic applications. The coherent correlation between experimental and docking outcomes positions the investigated compound as a promising candidate in the ongoing pursuit of effective antimicrobial agents.

**Table 9:**
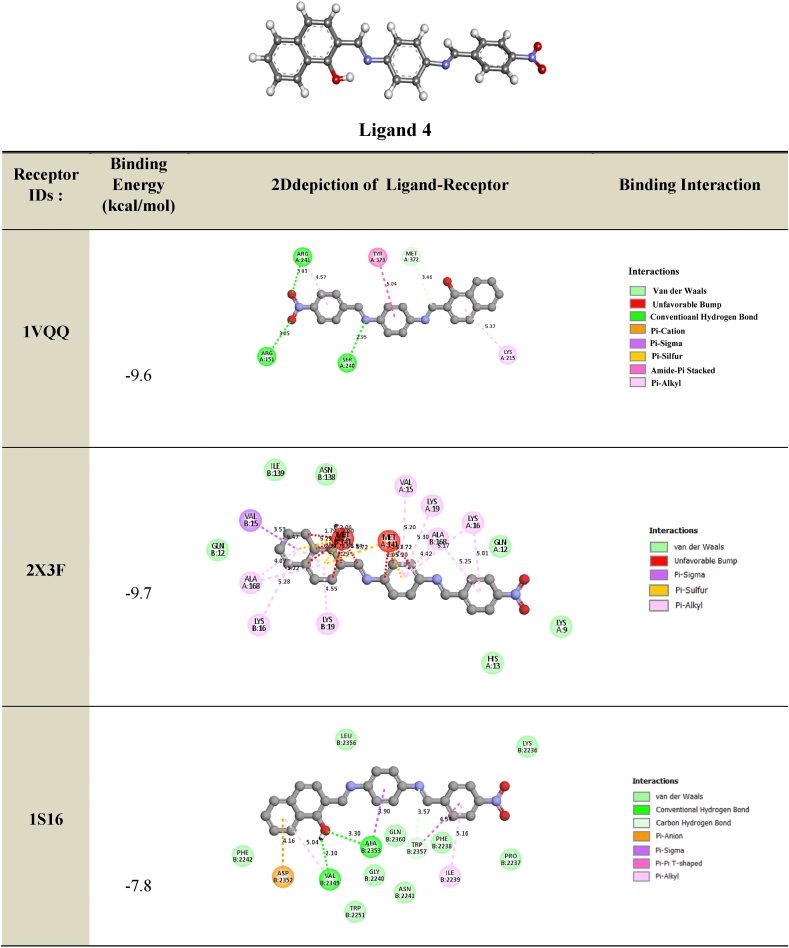
2D depiction of docking of ligand **4** with anti-*MRSA* targets (Binding Energy in kcal/mol).

## 4 Conclusion

In this work, we investigated a benzothiazole-based Schiff base. The solid state of the compound was examined by using elemental analysis, UVeVis, NMR and FT-IR studies. In order to understand and support experimental results, computational studies about structural, vibrational and electronic properties were performed at the B3LYP/6e311þþG(d,p) levels of theory. We observed that the calculated data are in good agreement with the experimental records. The new Schiff base ligand displayed intermediate antibacterial activities, with the highest activity observed against *C. Albicans fungi.* Furthermore, *in vitro* anticancer activities of the Schiff base ligand were observed towards SKOV3, PC-3, and HeLa tumor cells. Docking simulations were conducted to investigate the interactions between the 4NMN ligand and different amino acid proteins (receptors), and the relevant impact of the aromatic rings and oxygens on the biological activities of 4NMN was observed. A theoretical study was performed using the DFT/B3LYP/6-31+G(d) method to propose a suitable mechanism and predict the kinetics of the studied reaction and the stability of the desired products. UV absorption and emission spectra were experimentally determined in different media to investigate the optical properties of the ligand. The high fluorescence sensitivity in acidic media and the presence of tiny amounts of copper and chromium ions qualified 4NMN for use as a sensor for acidic medium, copper, and chromium ions. Regarding light characteristics, photon re-absorption was taken into account, and fluorescence effectiveness increased with increasing medium viscosity. TD-DFT calculations showed that the major transitions of the maximum wavelengths occurred at the frontier MO level.

## Sample availability

Samples of the compound are available from the authors.

## Data availability

The data used to support the ﬁndings of this study are available from the corresponding author upon request.

## CRediT authorship contribution statement

**Sadeq M. AlHazmy:** Data curation. **Mohamed Oussama Zouaghi:** Conceptualization. **Ahmed N. Al-Hakimi:** Data curation. **Thamer Alorini:** Formal analysis. **Ibrahim A. Alhagri:** Funding acquisition. **Youssef Arfaoui:** Investigation. **Rania Al-Ashwal:** Formal analysis. **Lamjed Mansour:** Investigation. **Naceur Hamdi:** Project administration.

## Declaration of competing interest

We wish to confirm that there are no known conflicts of interest associated with this publication and there has been no significant financial support for this work that could have influenced its outcome.
